# Development of the Human Arterial Valves: Understanding Bicuspid Aortic Valve

**DOI:** 10.3389/fcvm.2021.802930

**Published:** 2022-01-27

**Authors:** Deborah J. Henderson, Lorraine Eley, Jasmin E. Turner, Bill Chaudhry

**Affiliations:** Biosciences Institute, Newcastle University, Newcastle upon Tyne, United Kingdom

**Keywords:** arterial valve, semilunar valve, outflow tract cushions, human, development, remodelling, sculpting, bicuspid aortic valve

## Abstract

Abnormalities in the arterial valves are some of the commonest congenital malformations, with bicuspid aortic valve (BAV) occurring in as many as 2% of the population. Despite this, most of what we understand about the development of the arterial (semilunar; aortic and pulmonary) valves is extrapolated from investigations of the atrioventricular valves in animal models, with surprisingly little specifically known about how the arterial valves develop in mouse, and even less in human. In this review, we summarise what is known about the development of the human arterial valve leaflets, comparing this to the mouse where appropriate.

## Anatomy and Nomenclature of the Mature Arterial (Semilunar) Valves

**Figure 1:**
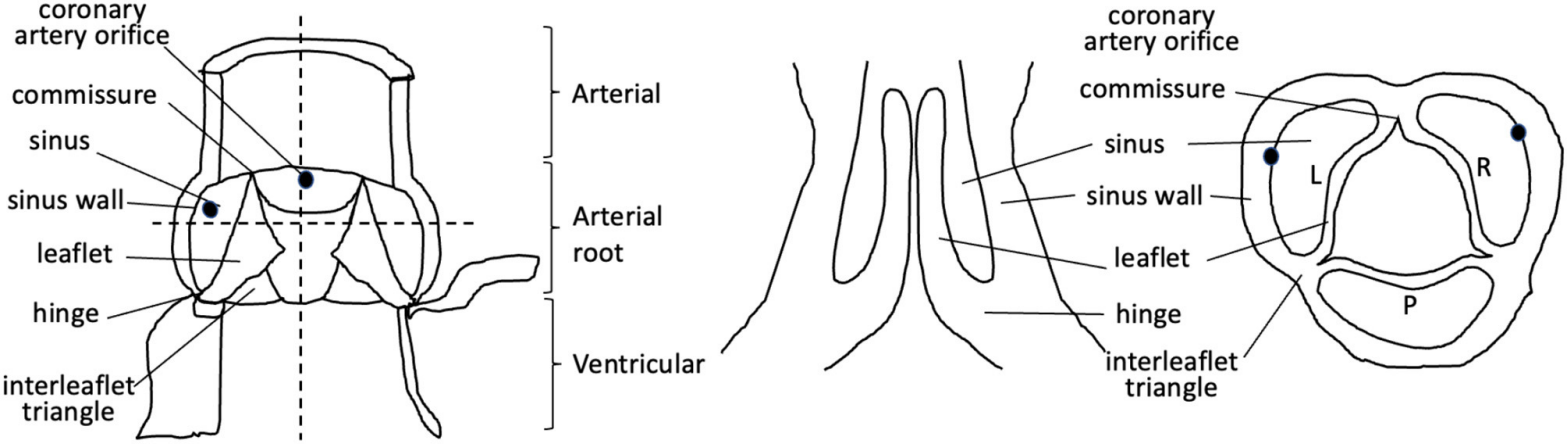
Cartoon illustrating the components of the mature aortic valve. Dotted lines denote longitudinal and cross-sections through the valve complex. L, left; R, right; P, posterior.

**Figure 2:**
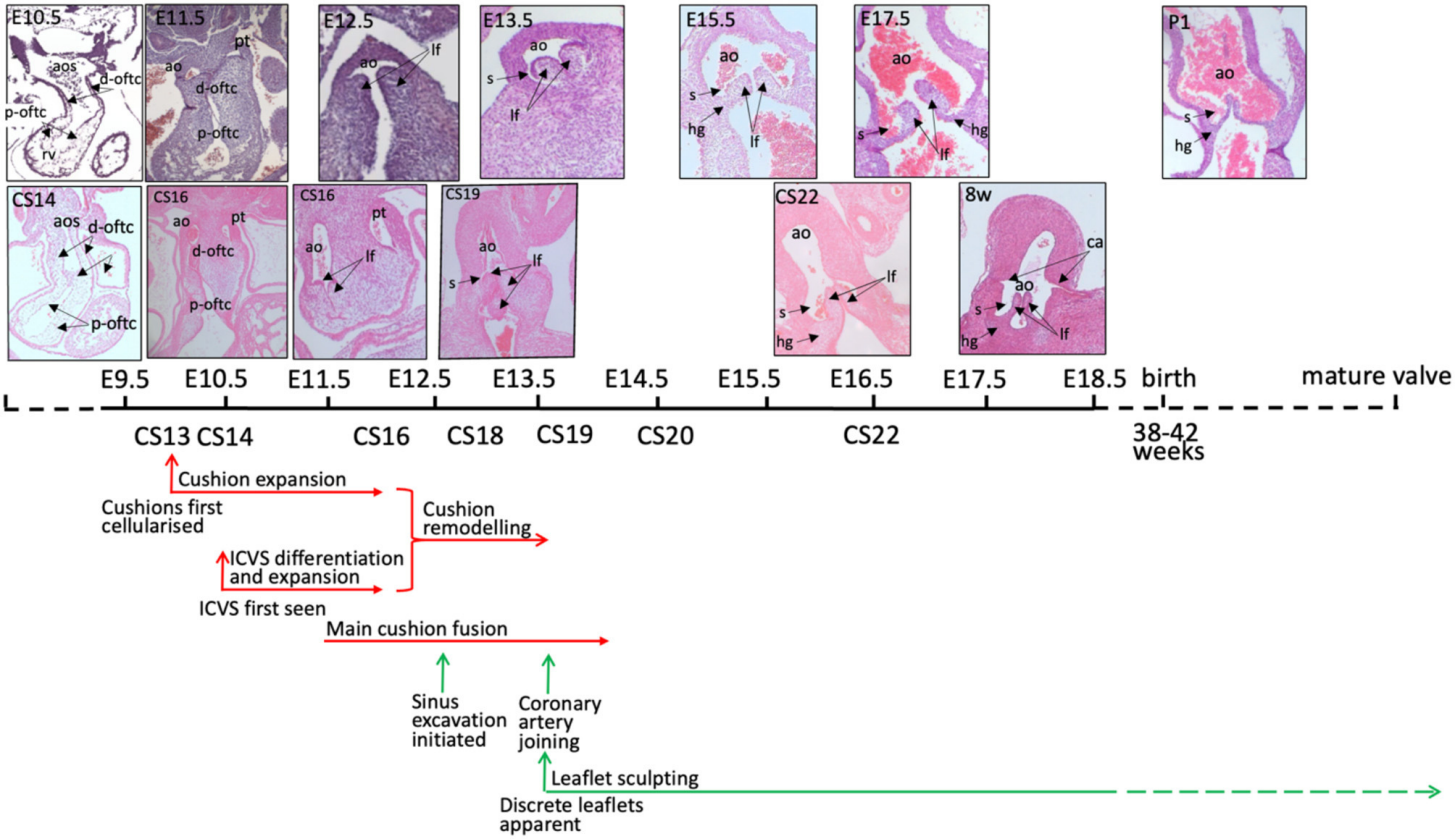
Timeline of mouse and human aortic valve development. Mouse and human outflow cushions and valves (stained with haematoxylin and eosin) are anatomically similar and follow a similar pattern of development. There is some variation between similarly Carnegie-staged human embryos such that, for example, CS16 embryos can be matched to mouse hearts at both E11.5 and E12.5. At 8 post-conception weeks of gestation, the human heart is similar to a mouse embryo shortly before birth, although it is still the first trimester. The human histology images were provided by the Joint MRC / Wellcome Trust (MR/R006237/1) Human Developmental Biology Resource (www.hdbr.org) (6). Mouse embryos were obtained from mice maintained according to the Animals (Scientific Procedures) Act 1986, United Kingdom, under project licence PPL 30/3876. All experiments were approved by the Newcastle University Ethical Review Panel. ao, aorta; aos, aortic sac; ca, coronary artery orifice; d-oftc, distal outflow tract cushions; hg, hinge; lf, leaflet; p-oftc, proximal outflow tract cushions; pt, pulmonary trunk.

Abnormalities of the arterial valves (sometimes known as the semilunar valves), including bicuspid aortic valve and leaflet dysplasia, are amongst the commonest congenital anomalies (1, 2). Although they may be asymptomatic at birth and thus missed during neonatal health checks, they do predispose to severe cardiovascular disease, including valve stenosis, regurgitation, calcification, and thoracic aortic aneurysm in later life (2). Thus, understanding their aetiology is of great interest. The arterial valves sit between the aorta and pulmonary trunk and their respective left and right ventricular outflow tracts in both human and mouse hearts, and are structurally very similar (3–5) (Figures 1, 2). These valves are complex structures that are composed of more than just the three moving leaflets. They also include the hinges that attach the leaflets to the wall, the commissures that are the points of apposition of the leaflets close to the wall, and the sinuses, which are the pockets that form between the leaflets and the wall. The regions of the wall that lie immediately upstream of the hinges (on the ventricular side) are called the interleaflet triangles (Figure 1). For the purposes of this review, we will refer to the coronary leaflets of the aortic valve (and their homologues in the pulmonary valve) as the right and left leaflet, and the non-coronary leaflet of the aortic valve as the posterior leaflet (its homologue in the pulmonary valve is the anterior leaflet). There have been several excellent reviews over recent years that have described in detail how the arterial valves develop based on studies in animal models (7–12). It is not our intention to extensively re-review the mouse, chicken, or zebrafish literature, but to instead focus on what is known directly from studies carried out with human material, relating this to key studies in animal models for perspective. The aim is to highlight some of the seminal work that has been done in the past, suggest what still needs to be done, and speculate on how this helps or hinders our understanding of one of the main congenital abnormalities of the arterial valves, bicuspid aortic valve (BAV).

## Endocardial to Mesenchyme Transition and Cushion Formation

The first and probably best understood process required for the development of the cardiac valves is the process of endocardial to mesenchyme transition (EndMT). This process, which has mostly been studied in mouse and chicken, has been reviewed extensively (7–12). In its primitive state, the heart tube consists of an outer layer of the myocardium, with an inner lining of the endocardium. Between these two cell layers is a layer of extracellular matrix (ECM), often known as cardiac jelly. This ECM is an essential component of the forming and mature valve leaflets, with the knockout of ECM components found in the early cushions, such as Hyaluronan and Versican, resulting in acellular cushions in mouse embryos [e.g. (13, 14)]. During the process of EndMT, endocardial cells change their epithelial-like phenotype into a mesenchymal one and migrate into the cardiac jelly to form primitive endocardial cushions (sometimes referred to as ridges). Whilst the regulation of the localisation and mechanisms of this complex process are beyond the scope of this review, some key points are relevant here. Cushions are first seen in the developing mouse outflow tract at about embryonic day (E)10, approximately 12 hours after they are seen in the atrioventricular canal. The cushions are cellularised between E10 and E11.5 by EndMT in the proximal region, which is regulated by Bone Morphogenetic Protein (BMP), Transforming Growth Factor beta (TGFβ), and Notch signalling [reviewed in (7–12)], and neural crest cells (NCCs) migrating into the distal cushions (described in more detail below). By E12.5, valve mesenchymal cells derived from both populations are found throughout the length of the cushions, thus contributing distally to the valve leaflets and proximally to the cushions that will give rise to the outlet septum (7–12).

Much less is specifically known about the early stages of cushion formation in the human heart. The outflow cushions are first apparent at the end of the 4th week of human gestation (CS10-13; 5-6mm crown-rump length; CRL. See Table 1 legend for explanation of staging used throughout this manuscript). Maron and Hutchins (1974) (16) saw cushions at approximately 33 days post conception (dpc; *CS15*) with Hutchins et al. (17) reporting the presence of outflow cushions in more than half of 32 dpc (*CS14*) hearts and all 33 dpc (*CS15*) hearts; these were recognised as taking on a spiralling configuration from their first appearance (18). There do not appear to be any studies directly investigating EndMT using cushion explant culture in human embryos. However, it was shown (19) that Nuclear Factor Of Activated T Cells 1 (NFATC1), which appears to repress EndMT in mice (20–22), was absent from nuclei of valve endocardial cells (VECs) of embryos at 4 weeks of gestation, but then appeared in the nuclei of these cells of embryos at 4–6 weeks (CS13-17) of gestation (19). This suggests that the absence of NFATC1 may allow VECs to undergo EndMT prior to 4 weeks of gestation. This correlates with when the outflow cushions first become apparent but, as in mice, may be important for repressing EndMT during the next phase when the cushions expand and septation occurs. Interestingly, the repressive effects of Vascular Endothelial Growth Factor A (VEGFA) on EndMT (23) also appear to be conserved (19).

**Table 1:** Staging criteria for human embryos.

**CS**	**Somite stage**	**Days post ovulation**	**Crown rump length (mm)**	**Morphological landmarks**
9	1–3	19–21	1.5–3.0	Head fold present
10	4–12	22	2.0–3.5	Neural tube closing. First and second branchial arches visible
11	13–21	24	2.5–4.5	Rostral neuropore closing. Otic placodes present. Optic vesicles formed.
12	21–29	26	3–5	Upper limb buds appear. Rostral neuropore closed. Caudal neuropore closing. Otic pits present. Three pairs of branchial arches visible.
13	30+	28	4–6	Embryo has C-shaped curve. Caudal neuropore closed. Upper limb buds flipper like. Four pairs of branchial arches visible. Lower limb buds appear. Otic vesicles present. Lens placodes distinct. Attenuated tail present.
14		32	5–7	Upper limbs paddle-shaped. Lens pits and nasal pits visible. Optic cups present.
15		33	7–9	Hand plates formed. Lens vesicles present. Nasal pits prominent. Lower limbs paddle-shaped. Cervical sinuses visible.
16		37	11–14	Foot plates formed. Pigment visible in retina. Auricular hillocks developing.
17		41	11–14	Digital rays clearly visible in hand plates. Auricle hillocks outline future auricle of external ear. Trunk beginning to straighten. Cerebral vesicles prominent.
18		44	13–17	Digital rays clearly visible in foot plates. Elbow region visible. Eyelids forming. Notches between the digital rays in the hand.
19		47.5	17–20	Limbs extend ventrally. Trunk elongating and straightening. Midgut herniation prominent. Prominent toe rays.
20		50.5	21–23	Upper limbs longer and bent at elbows. Fingers distinct but webbed. Notches between the digital rays in the feet. Scalp vascular plexus appears.
21		52	22–24	Hands and feet approach each other. Fingers free and longer. Toes distinct but webbed. Stubby tail present.
22		54	25–27	Toes free and longer. Eyelids and auricles or external ears more developed
23		56.5	28–30	Head more rounded and shows human characteristics. External genitalia still have sexless appearance. Distinct bulge still present in umbilical cord caused by herniation of intestines.

*(See http://hdbratlas.org/staging-criteria/carnegie-staging.html). Note on staging: The papers we have referenced use a range of staging methods, including Carnegie Stages (CS), days/weeks post conception (dpc/pcw), and crown-rump length (CRL). For comparative purposes and to aid understanding for the non-embryologist, these have been converted into days/weeks post conception. Where relevant, the original staging method follows in italics*.

## NCC, Outflow Tract Septation, and Valve Development

Outflow tract septation is intimately involved in arterial valve formation through shared progenitor cell lineages, cushions, and timing of events. These progenitor lineages include EndMT-derived cells (which in the outflow tract originate in the second heart field; SHF) and NCCs. The NCCs are a progenitor population that play a crucial role in the process of outflow tract septation; in their absence, septation does not occur [reviewed in (24–26)]. Whilst NCCs form smooth muscle cells in the developing walls of the great arteries and are important in cardiac innervation, it may be that their role in outflow septation is mainly as a ‘filler,’ acting to bulk out the outflow cushions to bring them into contact and thus allow fusion; the majority of NCCs in the proximal part of the cushions die by apoptosis shortly after septation occurs (12, 27). It has been recognised that outflow tract septation cannot be initiated before the formation of the 6th pharyngeal arch arteries (28), which does not occur until approximately 32 dpc [*CS14* or 6–8 mm; (29)]. The aorta and pulmonary blood flows are separated by around the end of the 5th week of gestation (*CS15-16*; 9 mm CRL) (16), with the initiation of outflow septation apparent in most 37 dpc embryos (*CS16;* 11–14 mm CRL) (17). Kramer (15) showed that this septation produces the precursors of the right and left leaflets of the forming arterial valves, with the parietal main outflow cushion contributing to the left leaflets of the aortic and pulmonary valve and the septal cushion contributing to the right leaflets. Notably, at least in the mouse, both EndMT-derived cells and NCCs are abundant in the left and right leaflets of the aortic and pulmonary valves at comparable and later stages of development (30, 31). Fusion of the main cushions separates the channels of the aorta and pulmonary trunk and subsequent separation of the aorta from the pulmonary trunk in a plane perpendicular to the line of cushion fusion results in the formation of two, free-standing arterial trunks with their distinct valves. The aortic and pulmonary valves are approximately lateral to one another at 37 dpc (*CS16*), with the pulmonary valve only slightly more cranial. This is much exaggerated by 48 dpc (*CS19*) with, similarly to mouse, the pulmonary valve positioned more superior and anterior (16) (anterior and ventral in the mouse).

Although it is accepted that NCCs are crucial for human outflow tract development as they are in birds and mice [reviewed in (24, 25)], there is limited information available, at least in part because of the difficulty in labelling this progenitor cell population in the human heart. It has been shown, using AP2α as a marker, that NCCs are found within the distal outflow cushions at 33 dpc [*CS15*; (32)]. This expression was downregulated by 37 dpc (*CS16*) of development and thus could not be used to track them within the developing arterial valves. However, the characteristic condensed mesenchyme within the outflow cushions, which is shown to be made up of NCCs in the mouse, is apparent in the human outflow cushions from approximately 32 dpc (*CS14*) (33).

## Formation of Intercalated Leaflet Valve Swellings

The intercalated leaflet valve swellings (ICVSs; sometimes referred to as intercalated valve cushions) that are the precursors of the anterior and posterior leaflets of the arterial valves were first noted in human embryos (15) and are seen clearly in cross sections through the outflow tract at 33 dpc (*CS15;* 9 mm *CRL*) as lateral bulges within the outflow wall; they have since been described in the mouse at comparable stages of development (E10.5-E12.5). Kramer (15) recognised during his original observations that the ICVSs contribute solely to the valve leaflets and do not play roles in outflow tract septation. Moreover, he recognised that they are distinct from the main outflow cushions; this has since been confirmed by studies in mouse (31). The ICVSs of human embryos were also discussed by Thompson et al. (34) who described them as midline cellular condensations close to the myocardial rim, although he did not recognise their role in valve formation. He noted, however, that the ‘condensations’ were less distinct in other species, including the rat and chicken (34). Of note, care should be taken when using the word ‘condensations’ with respect to the ICVSs, as this could lead to confusion with the more frequently referred to condensations of NCCs that occur in the main outflow cushions (see above). More recently, and after a gap of more than 20 years, several authors have recognised the ICVSs in mouse and human embryos and have begun their molecular characterisation (31, 32, 35, 36). This has shown that they lie at the boundary between the SHF-derived myocardium (proximally) and SHF-derived smooth muscle cells (distally) within the walls of the outflow vessels at E10.5-E12.5 (human 28–33 dpc; *CS13-15*) (31, 35). In terms of their cellular origin, it has been shown that whilst there are small numbers of EndMT-derived and NCC-derived cells within the ICVSs, the majority of cells at these early stages express Isl1, a marker of undifferentiated SHF-derived cells, suggesting that they have a different origin (directly differentiating from SHF progenitors) to the majority of cells in the main outflow cushions that are formed by EndMT and the immigration of NCCs (31, 32).

## Cushion Expansion to Form Arterial Valve Primordia

**Figure 3:**
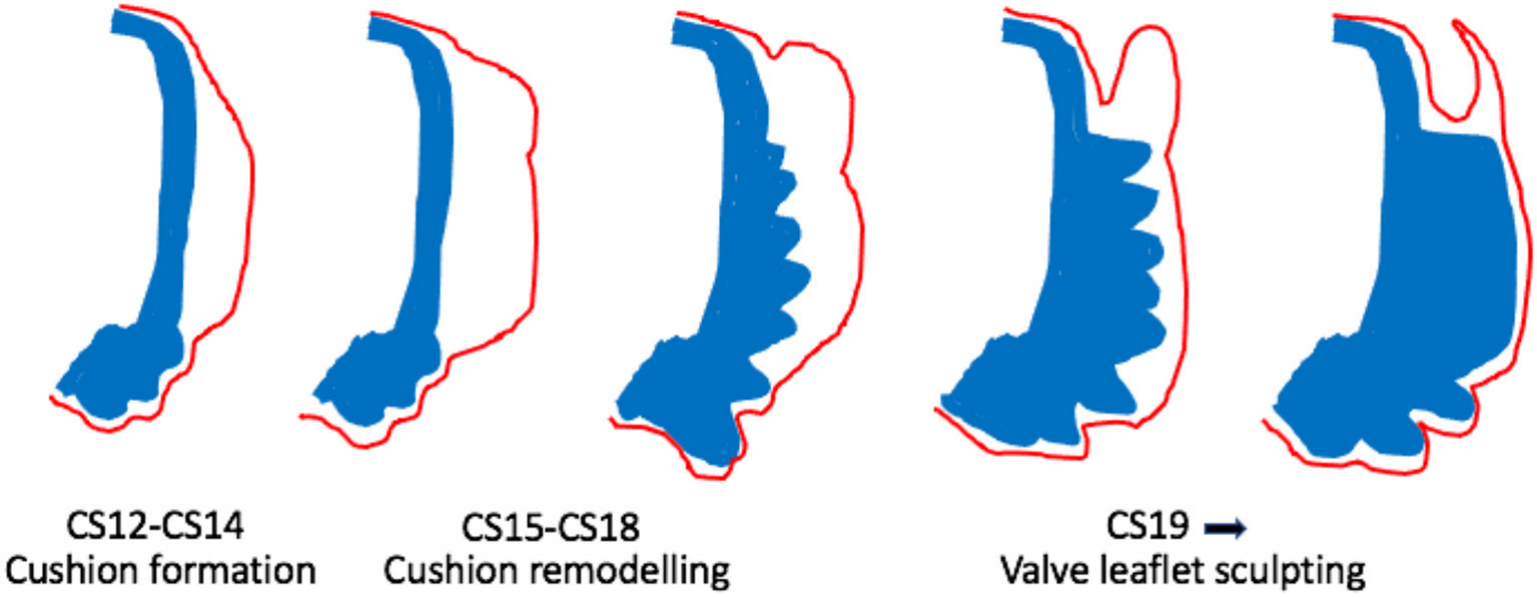
Cartoon illustrating morphological changes in outflow cushion/valve formation. The outflow cushions first appear at CS12-14 (equivalent to mouse E10-E11). Between CS15-18, the distal parts of the cushions remodel to form the valve precursors. From CS19 onwards, these valve precursors are sculpted to produce the thin valve leaflets with the sinus behind. Blue denotes the outflow wall. Red denotes the endocardium.

Once the relevant progenitor lineages have contributed cells to the mesenchyme in the mouse heart, they undergo a period of proliferation that results in rapid cushion growth (as shown in Figures 2, 3), with too much [e.g. (37, 38)] or too little [e.g. (39, 40)] cell division associated with defects in the leaflets as development proceeds. More than 60% of outflow cushion mesenchymal cells express proliferation markers during the 4th week of human development, with the levels falling gradually to <20% after the 10th week of gestation (19). RNASeq analysis suggested that this might be regulated by MYC proto-oncogene (MYC), a known regulator of cell cycle progression. Thus, as in the mouse, there is rapid proliferation leading up to the period when cushions remodel into valve leaflets (see below) (19).

As well as its early roles in supporting EndMT, ECM also appears to be important for remodelling of the cushion/valve mesenchyme. Knockout of the ECM component Perlecan in mice results in excessive numbers of cells within the outflow cushions, resulting in cushion hyperplasia and dysplastic arterial valve leaflets (41). Similarly, Versican, which is essential for early cushion formation, also plays an essential role during later remodelling stages of cushion to valve morphogenesis. If Versican cleavage is impaired by knockout of the matrix metalloprotease ADAMTS5, then the arterial valve leaflets become dysplastic, with abnormal deposition of other matrix components including Collagen and Elastin (42). In the human heart, CD44 (Hyaluronan receptor) has been found in cushion mesenchyme cells at 4 weeks of gestation (approximately *CS13*) and slightly later in valve endocardium (19). Elastin staining using specific antibodies was seen from 7 weeks of gestation (*CS19-20*) in the developing arterial valve leaflets (although at lower levels than in the arterial wall) and already showed evidence of stratification, being more abundant on the ventricular side of the forming leaflets (43). Elastin expression declined with gestational age. RNASeq showed enrichment for a number of other microfibril-associated proteins, including Fibronectin, Fibrillin 2 and 3, Fibulin 2,4 and 5, LOX, and Emilin, in first trimester valve tissue, with Fibronectin and Fibrillin 1–3 found by immunohistochemistry from 4 weeks (43).

Recently, there has been much interest in the potential role of cilia in the development and maintenance of valve leaflets, largely because of the association between mutations in cilia-associated genes and human valve defects [e.g. (44)]. Primary cilia are small projections of the membrane with a microtubule core, which are implicated in cell signalling and mechano-sensing (45). Currently, there do not appear to be any published studies characterising cilia in human embryonic or foetal arterial valve leaflets; this is an area that would benefit from further work.

## Valve Sculpting

Once the cushions have formed and outflow tract septation is underway (from approximately E12.5 in mouse; *CS16* in human), the distal parts of the main cushions remodel to form the arterial valve leaflets and are distinct from the proximal cushions that will remodel to form the outlet septum of the ventricular outflow tracts (Figures 2, 3). During this process of leaflet remodelling, the details of which remain unclear, the bulky, primitive valve precursors (cushions and ICVSs) are sculpted into thin fibrous leaflets, connected to the vessel wall by hinges that delineate the sinuses (Figure 3). A number of mouse mutants have been described as having thickened, abnormally shaped (dysplastic) leaflets [for example (41, 42, 46–49)]. The leaflets in these mutants are characterised by having excessive cell numbers and/or abnormalities in the ECM; if this leads to the cushions being pushed against one another, it can result in BAV (42, 49). However, in most cases, the pathomechanisms leading to valve dysplasia are unclear. Thus, these sculpting stages of arterial valve development remain the least well-understood but may be the most relevant to human disease.

Since the arterial valve leaflets form from the distal ends of the outflow cushions (15, 50), it may be possible to determine where they will form from as early as 33 dpc (*CS15*) as the lumen narrows and the overlying endocardium takes on a distinct cobble-stone appearance. However, at 37 dpc (*CS16*), the mesenchyme of the distal cushions, where the leaflets will form, swells relative to the more proximal regions of the cushions (33). The first appearance of valve structures from the cushions is at approximately 41 dpc (*CS17*), with the sinuses first apparent as shallow depressions between the arterial wall and the developing valve (16) (Figures 2, 3). At this stage, it was also observed that the VECs on the arterial side of the leaflet are cuboidal whereas those on the ventricular side are flattened (16), suggesting responses to differential blood flow patterns. This was later confirmed using molecular markers [CD31; (19)]. At this stage, the forming valves have a considerable length within the middle part of the septating outflow tract, coinciding with the boundary between the arterial and ventricular components (33). By around the 50th day of gestation (*CS20*), the sinus walls are becoming apparent but are still surrounded by a cuff of the myocardium, whilst by 52 dpc (*CS21; 23mm CRL*), the leaflets are short and thick with only a slit-like sinus. At the same time, the cellular density of the leaflets is increasing. By the end of the 8th week of gestation (*CS23*), the leaflets appear longer and are more delicate (16) (see Figures 2, 3). The leaflets continue to become more refined as development proceeds.

## Maturation of the Arterial Valve Leaflets

At the same time as the leaflets are sculpting, they are also maturing in respect to their cellular and extracellular components. Mature valve leaflets contain valvular interstitial cells (VICs) that are derivatives of the cushion mesenchyme. These VICs reside within three layers, characterised by distinct ECM profiles: the fibrosa (on the arterial side of the leaflet), composed mostly of Collagens; the spongiosa, made up mostly of Proteoglycans; and the ventricularis, which contains Elastin fibres. The leaflets are also covered by a specialised endocardial layer (VECs) (51). Animal studies have shown that abnormalities in, for example, matrix remodelling enzymes can disrupt the stratification of the valve leaflets during development (42, 51), whereas in other models [e.g. (51, 52)], the leaflets are initially stratified, but become disorganised and myxomatous as the animals age. The timing of leaflet stratification in human embryos is rather unclear as in the limited studies that have been carried out, there are large gaps between stages. One study suggested that the arterial valve leaflets lose their homogeneous character in the 60 mm CRL foetus (approximately 12 pcw), with Collagen fibrils apparent in the proximal parts of the leaflets and in the subendocardial region of their ventricular side (16). Analyses of more mature valves were carried out in this study (16), however, CRL was used to determine the maturity of the samples, which is not generally considered to be reliable after 80 mm due to a high degree of variation between foetuses. Thus, the precise staging of larger foetuses from this study has to be interpreted with caution. Collagen fibres increased in the leaflets of the 90 mm foetus (approximately 14 pcw) and by 100 mm (approximately 14.5 pcw), a distinct subendocardium, with elastic fibres in the ventricular side, was observed. Collagen continued to increase in the 100 mm and 125 mm foetus (approximately 16 pcw). The valve leaflets began to take on a mature structure in the 150 mm foetus with a dense collagenous middle layer and looser connective tissue on the ventricular side of the leaflets (16). Analysis of human hearts from 14 gestational weeks (using Movat's pentachrome staining) indicated that, at this earliest stage, the valves were homogeneous with no clear layers apparent. At these stages, the valves were mainly composed of Proteoglycans, with limited Collagen and Elastin (52). By 20 weeks, a bilaminar structure was observable, but the mature trilaminar structure was not apparent until 36 weeks of gestation. It was also noted that VIC density decreased in the 2nd and 3rd trimesters, and this was matched by a decrease in proliferation from approximately 26% in the second trimester, to only 6% in the third trimester; by this stage, most of the proliferation was close to the arterial side of the leaflets. The second trimester foetal valve leaflets had a myofibroblast-like appearance and expressed high levels of proteolytic enzymes such as Matrix metalloproteases (MMPs); these decreased with advancing gestational age (53).

## The Appearance of the Coronary Arteries

Coronary arteries were seen in the left and right sinuses of the aortic valve during the 6th week of human development *(CS18*) (17). This was confirmed recently (33) with data showing that the left coronary artery is first seen at CS18, whereas the right coronary artery was only seen a few days later at CS19. Notably, it has been suggested that apart from the association of the coronary arteries with the right and left leaflets of the aortic valve only, the aortic and pulmonary valves appear morphologically similar until birth (16).

## Arterial Valve Malformations – Genetic Causes?

Congenital malformations of the arterial valves are very common and include valves with abnormal numbers of leaflets, including unicuspid, bicuspid, and quadricuspid variants, and valve leaflet dysplasia, in which the leaflets are typically thickened and/or shortened (1, 2). In the case of BAV, which is by far the most common abnormality, a similar range of leaflet patterns has been observed in human and mouse, including apparent fusions between the left and right and right and posterior (non-coronary) leaflets; fusions between the left and posterior leaflets are rare in humans (1, 2). Moreover, it has been suggested that BAV can also result from the absence of a leaflet, presumably the posterior leaflet derived from the ICVS, as an absence of one of the main outflow cushions would prevent outflow tract septation (12, 31, 54). A well-recognised feature of bicuspid valves is the presence or absence of a raphe, presumed to represent a fusion seam, which in some cases is associated with a notch or an asymmetry suggesting a third leaflet formed during development, but fused with one of the others. Studies in animal models have suggested that leaflet fusion occurs because the cushions or leaflets are pushed against one another resulting in their fusion, either because they are hyperplastic or misplaced, with the raphe being what remains of the endocardium at the fusion seam e.g. (49, 54). Although it is commonly stated that 85–90% of human BAVs have a raphe (1, 2), recent studies suggest that as many as 25% may have no raphe (55). This implies that in these cases, either the fusion seam is completely remodelled and cannot be seen, or the leaflet primordium may have been missing throughout development. All of these abnormalities predispose the valves to stenosis and/or regurgitation, and to degeneration in later life. Interestingly, there appear to be differences in the patterns of abnormalities seen in populations from different ethnic groups. For example, studies from Europe and the United States have suggested that approximately 85% of non-syndromic patients with BAV present with a common left-right leaflet (1, 2). In contrast, studies from Japan and South Korea suggest that the left-right pattern may be found in <60% of East Asian patients with BAV, with a common right-posterior leaflet increasing in prevalence in this population (56, 57). Common left-posterior leaflets are rare in any population. Although this requires further investigation, this suggests that there may be a genetically inherited predisposition to particular types of malformation. Recent studies have also suggested that genetic syndromes can be associated with different patterns of leaflet fusion. For example, whilst Turner and DiGeorge syndromes most commonly present with a common left-right leaflet, Down syndrome is associated with a common right-posterior leaflet (58). Together, these data suggest that there is likely to be a genetic element to leaflet patterning.

There have been several large (and small) studies that have sought to establish the genetic causes of BAV and aortic stenosis in the human population [for example (59–67)]. Despite some success [most notably in the case of ROBO4; (68)], many of these studies have taken a targeted-sequencing approach based on data from studies of animal models and/or filtered the large numbers of gene variants obtained from exome sequencing based on whether the gene is known to play a role in heart/valve development. Thus, these studies are heavily reliant on the quality of the data from previous genomic studies and from cardiovascular developmental biology using animal models.

**Figure 4:**
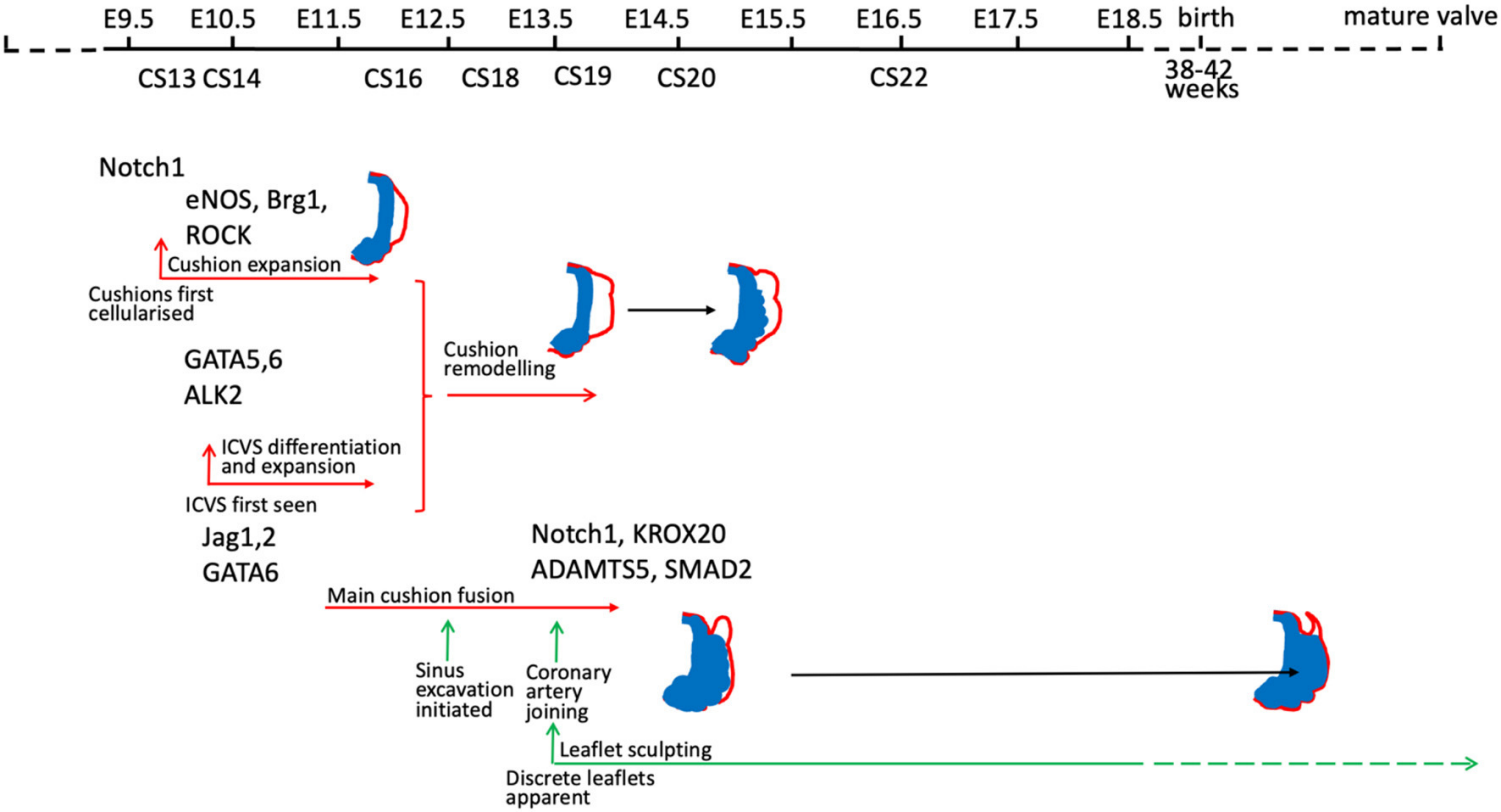
Timeline illustrating the timing of gene activity (based on developmental process disrupted) in mouse models of BAV. Mouse models included are those for which mechanistic studies have been carried out to understand why BAV develops. Notch 1 (31, 71); eNOS (69, 76, 77); Brg1 (78); ROCK (30); GATA5 (70); GATA6 (63); ALK2 (72); Jag1/2 (31, 71); Krox20 (49), ADAMTS5/Smad2 (42).

There are many good examples of mouse gene knockouts that result in valve dysplasia and/or BAV [for example (30, 31, 42, 49, 63, 68–73)] and also in other species such as the Syrian hamster (54, 74, 75). These animal models have given essential insights into how disruption of different developmental mechanisms, genes, and signalling pathways can lead to different types of BAV and valve dysplasia (Figure 4). For a comprehensive overview of this topic, we recommend several excellent reviews where these have been summarised (12, 54, 79, 80). It should be noted that only rarely (68) has the mouse been used to model human BAV gene variants, confirming their pathogenicity, and thus it remains unclear in the majority of cases whether the gene variants observed are directly and specifically causative for BAV.

Although each of the different patterns of BAV has been reported in mouse models, there appears to be an under-representation of the left-right fusion pattern relative to the human population, and an over-representation of the cases where there appears to be an absence of the posterior leaflet, with no visible raphe in the remaining leaflets. Some of these differences may be explained by a lack of detailed description of the tiny mouse leaflets, where a raphe may be difficult to see. It could also be that the nature of most mouse models, where genes are ‘knocked out’ and are therefore non-functional may lead to different effects on cell behaviour. Point mutations may alter the function of the associated genes in more subtle ways, perhaps altering interactions with binding partners leading to gain of function rather than loss of function phenotypes. Alternatively, most human BAV series involve adult patients and it may be that this is not fully representative of the full spectrum of anomalies. For example, a study of babies under 3 months of age with critical aortic stenosis revealed different leaflet and commissure patterns to those of older children (81). Whether this is a maturity issue or reflects a failure of survival of babies with the severest phenotypes is not clear, but it does suggest that an analysis of only adults may miss some phenotypic variations. It is also possible that there are biological differences between mouse and human arterial valve development and anatomy. Some minor differences are apparent (Figure 2), but whether these are enough to explain the reported differences in aortic valve phenotypes between mouse and human remains unclear.

There is a great interest in the idea of using zebrafish to model human BAV gene variants, despite the zebrafish aortic valve naturally having only two leaflets; there is no pulmonary valve in fish as there are no lungs. There would be enormous advantages of using the zebrafish to model BAV variants, as it develops externally, is easy to manipulate physically and genetically, and lack of dependence on a functional cardiovascular system for the first 5 days of its development, coupled with its transparency as a larva, makes it ideal for live imaging of the developing heart and for investigating the more genetically intractable effects of, for example, blood flow on valve development (82). However, this major anatomical difference between fish and mammals means that it is not easy to directly correlate abnormal phenotypes in the zebrafish valve with the human phenotype. Despite this, aortic stenosis and/or regurgitation across the aortic valve has been used as supportive evidence for gene variant significance (68, 83, 84). The use of zebrafish to model BAV is also currently hampered by a lack of clarity about whether the zebrafish arterial valve develops in the same way as the mammalian valve. For example, do the same progenitor lineages contribute to the aortic valve in mammals and fish, and does EndMT plays a crucial role in zebrafish as it does on mouse? Elucidation of these issues will improve our understanding of the key features of valve development and will help to establish whether zebrafish can be a useful model for understanding how gene variants in human patients can lead to the well-recognised pathology.

Much is known about arterial valve development from animal models, although there are still some areas, particularly relating to the sculpting of the leaflets and the maturation of their supporting structures, that remain to be clarified. In terms of understanding the extent to which knowledge from animal studies can be applied to human arterial valve development, we still do not have a detailed timeline of how the arterial valves develop in the human embryo/foetus, particularly in the period from 8 to 40 weeks of gestation. Although the samples are limited, it will be important to establish when the key events take place and to establish the maturity of the human foetal and neonatal arterial valves relative to what we see in rodents. In terms of morphogenetic mechanisms, remarkably little is known specifically about these processes in human embryos. Although direct extrapolation from mouse to human is routine, we still do not know if cardiac progenitor populations make the same contributions in mouse and human. This is hampered by the difficulties in carrying out lineage tracing experiments in human embryos, although this could be overcome by the development of specific markers for differentiated NCCs and EndMT-derived cells. Alternatively, tracking somatic mutations as a form of lineage tracing is becoming a reality and may solve the issue (85). Extending from this, we do not know whether the same mechanisms that seem to underpin the patho-development of BAV in mice, for example, hypercellularity of the cushions/leaflets or absence of the ICVS, are similarly of relevance in humans. Thus, whilst it is likely that the basic mechanisms are similar, there may be crucial differences that limit the usefulness of animal models for studying both normal and abnormal valve development and disease. Whilst the possibility of offering genetic counselling for isolated BAV may seem some way off, and anyway may not be considered appropriate for a malformation that is frequently asymptomatic for much of life, it would be useful to understand the link between BAV and adult cardiovascular disease. This might allow us to stratify patients with BAV into groups who would be likely to go on to develop calcified leaflets or thoracic aortic aneurysms, perhaps based on the leaflet pattern or the nature of the progenitor lineage that was disrupted, and those in which this is unlikely. This would allow targeted drug interventions and perhaps influence surgical decisions about whether to replace the aortic valve with the patient's own pulmonary valve (as in the Ross procedure), rather than a porcine, synthetic, or metallic valve. It is only with further analysis of scarce human embryos that we will properly be able to address these important issues.

## Author Contributions

DH and BC wrote, reviewed, and revised the manuscript. LE and JT produced the histological sections and reviewed the document. All authors contributed to the article and approved the submitted version.

## Funding

This work was supported by the British Heart Foundation Programme Grant RG/19/2/34256 and MRC/Wellcome Trust (MR/R006237/1).

## Conflict of Interest

The authors declare that the research was conducted in the absence of any commercial or financial relationships that could be construed as a potential conflict of interest.

## Publisher's Note

All claims expressed in this article are solely those of the authors and do not necessarily represent those of their affiliated organizations, or those of the publisher, the editors and the reviewers. Any product that may be evaluated in this article, or claim that may be made by its manufacturer, is not guaranteed or endorsed by the publisher.
